# Prediction of the Effects of Empagliflozin on Cardiovascular and Kidney Outcomes Based on Short-Term Changes in Multiple Risk Markers

**DOI:** 10.3389/fphar.2021.786706

**Published:** 2022-01-25

**Authors:** Sok Cin Tye, Sieta T. de Vries, Christoph Wanner, Petra Denig, Hiddo J. L. Heerspink

**Affiliations:** ^1^ Department of Clinical Pharmacy and Pharmacology, University Medical Centre Groningen, Groningen, Netherlands; ^2^ Division of Nephrology, Department of Medicine, Würzburg University Clinic, Würzburg, Germany

**Keywords:** empagliflozin, diabetes, risk markers, cardiovascular outcomes, kidney outcomes

## Abstract

**Aims:** The EMPA-REG OUTCOME trial demonstrated that the sodium-glucose cotransporter-2 inhibitor (SGLT2) empagliflozin reduces the risk of cardiovascular (CV) and kidney outcomes in patients with type 2 diabetes. We previously developed the parameter response efficacy (PRE) score, which translates drug effects on multiple short-term risk markers into a predicted long-term treatment effect on clinical outcomes. The main objective of this study was to assess the accuracy of the PRE score in predicting the efficacy of empagliflozin in reducing the risk of CV and kidney outcomes.

**Methods:** Short-term (baseline to 6-months) changes in glycated hemoglobin (HbA1c), systolic blood pressure (SBP), urinary-albumin-creatinine-ratio (UACR), hemoglobin, body weight, high-density-lipoprotein (HDL) cholesterol, low-density-lipoprotein (LDL) cholesterol, uric acid, and potassium were determined among 7020 patients with type 2 diabetes and established CV disease in the EMPA-REG OUTCOME trial. The beta-coefficients, derived from a Cox proportional hazards model in a pooled database consisting of 6355 patients with type 2 diabetes, were applied to the short-term risk markers in the EMPA-REG OUTCOME trial to predict the empagliflozin-induced impact on CV (defined as a composite of non-fatal myocardial infarction, non-fatal stroke, or CV death) and kidney (defined as a composite of doubling of serum creatinine or end-stage kidney disease) outcomes.

**Results:** Empagliflozin compared to placebo reduced HbA1c (0.6%), SBP (4.2 mmHg), UACR (13.0%), body weight (2.1 kg), uric acid (20.4 μmol/L), and increased hemoglobin (6.6 g/L), LDL-cholesterol (0.1 mmol/L) and HDL-cholesterol (0.04 mmol/L) (all *p*<0.01). Integrating these effects in the PRE score resulted in a predicted relative risk reduction (RRR) for the CV outcome of 6.4% (95% CI 1.4–11.7), which was less than the observed 14.7% (95% CI 1.3–26.4%) RRR. For the kidney outcome, the PRE score predicted a RRR of 33.4% (95% CI 26.2–39.8); the observed RRR was 46.9% (95% CI 26.8–61.5). In a subgroup of 2,811 patients with UACR ≥30 mg/g at baseline, the PRE score predicted RRR was 40.8% (95% CI 31.2–49.1) vs. the observed RRR of 40.8% (95% CI 12.4–60.0) for the kidney outcome.

**Conclusions:** Integrating multiple short-term risk marker changes in the PRE score underestimated the effect of empagliflozin on CV and kidney outcomes, suggesting that the currently used risk markers do not fully capture the effect of empagliflozin. In patients with increased albuminuria, the PRE score adequately predicted the effect of empagliflozin on kidney outcomes.

## Introduction

Major cardiovascular (CV) outcomes trials of sodium-glucose cotransporter-2 (SGLT2) inhibitors, developed as anti-hyperglycemic drugs for the treatment of type 2 diabetes, have consistently demonstrated that these drugs reduce the relative risks of CV outcomes and slow the progression of kidney function decline in patients with type 2 diabetes at high CV risk (McGuire et al., 2021). The EMPA-REG OUTCOME trial was the first published trial to demonstrate the benefit of an SGLT2 inhibitor, empagliflozin, for CV and kidney protection (Barnett et al., 2014; Zinman et al., 2015).

The efficacy of SGLT2 inhibitors in preventing CV outcomes and delaying diabetic kidney disease progression is unlikely explained by improvement in glycemic control alone. Head-to-head comparison of the SGLT2 inhibitor canagliflozin with the sulfonylurea derivative glimepiride showed that at equal glycemic control, canagliflozin reduced the rate of kidney function decline (Heerspink et al., 2017). In patients without diabetes, the SGLT2 inhibitor dapagliflozin did not reduce glycated hemoglobin (HbA1c) but significantly reduced the risk of CV events and kidney failure (Persson et al., 2021).

Numerous studies have shown that SGLT2 inhibitors exert additional effects beyond their effects on HbA1c. These effects can contribute to long-term CV and kidney protection (van Bommel et al., 2017; Vallon and Verma, 2021). For example, SGLT2 inhibitors reduce glomerular hyperfiltration, systolic and diastolic blood pressure, improve tubular oxygen tension, and reduce body weight, uric acid, and albuminuria (Heerspink et al., 2016; Heerspink et al., 2019). Experimental and clinical studies have shown anti-inflammatory and anti-fibrotic effects, reflected by reductions in interleukin-6 and monocyte chemoattractant protein-1, although such effects may be secondary to improvement in glycemic control (Vallon et al., 2014; Tahara et al., 2017; Mancini et al., 2018).

Since SGLT2 inhibitors exert multiple effects on CV and kidney risk markers, we hypothesized that integrating the effects of empagliflozin on multiple CV or kidney risk factors may be able to better predict the effect of empagliflozin on these clinical outcomes than focusing on changes in a single risk marker. We have previously developed a multivariable drug efficacy prediction algorithm (i.e., the multiple Parameter Response Efficacy (PRE) score) to predict long-term drug effects on CV and kidney outcomes. Specifically, the PRE score was used to predict the efficacy of angiotensin receptor blockers, the endothelin receptor antagonist atrasentan, and the glucagon-like peptide-1 receptor agonists exenatide and liraglutide in patients with type 2 diabetes at high risk of CV disease or with established chronic kidney disease (Smink et al., 2014a; Schievink et al., 2015; Schievink et al., 2016; Idzerda et al., 2020; Tye et al., 2021) The objective of this study was to assess the accuracy of the PRE score in predicting the effect of the SGLT2 inhibitor empagliflozin on CV and kidney outcomes in patients with established CV disease who participated in the EMPA-REG OUTCOME trial. Secondly, we used the PRE score to prospectively predict the composite kidney and CV death outcome for the ongoing EMPA-KIDNEY trial.

## Materials and Methods

### Study Design and Population

The PRE score was used to estimate the effect of empagliflozin on CV and kidney outcomes. The PRE score is a flexible algorithm that is intended to be used to any population or drug by fitting the beta coefficients in the underlying Cox proportional hazards model to the appropriate set of risk markers in an independent population. For this specific study, the relationships between risk markers and CV, or kidney outcomes were established at baseline in a background population derived from the ALTITUDE, RENAAL and IDNT trials, clinical trials conducted in patients with type 2 diabetes at high risk of kidney events or with established CV disease (Supplementary Table S1). This combined database consisted of 6355 type 2 patients in whom 794 (12.5%) CV outcomes and 1129 (17.8%) kidney outcomes were recorded. The designs and primary results of the individual trials have been previously published (Brenner et al., 2001; Lewis et al., 2001; Parving et al., 2012).

The estimated risk relationships were subsequently applied to all patients from the EMPA-REG OUTCOME trial to assess the accuracy of the PRE score. Since patients with elevated albuminuria are at higher risk of CV and kidney outcomes compared to patients in whom albuminuria is in the normal range, we performed a subgroup analysis in patients with urinary-albumin-creatinine-ratio (UACR) >30 mg/g. The PRE score predictions were compared to the observed CV and kidney outcomes in the EMPA-REG OUTCOME trial. We also predicted the effect of empagliflozin in the EMPA-KIDNEY trial (NCT03594110), an ongoing clinical trial designed to assess the effect of empagliflozin on kidney outcomes for which the outcome of the trial is not yet known. To this end, we selected patients from the EMPA-REG OUTCOME trial who met the inclusion criteria of the EMPA-KIDNEY (NCT03594110) trial. We applied the PRE score to the subgroup of patients to predict the effect of empagliflozin on the primary endpoint in the EMPA-KIDNEY trial (Herrington et al., 2018).

### Risk Markers Selection

Variables measured in the intention-to-treat population of the EMPA-REG OUTCOME trial at baseline and after 6 months which were previously identified as risk markers for CV and kidney outcomes were used, i.e., glycated hemoglobin (HbA1c), systolic blood pressure (SBP), UACR, hemoglobin, body weight, high-density-lipoprotein (HDL) cholesterol, low-density-lipoprotein (LDL) cholesterol, uric acid, and potassium (K). The 6 month timepoint was chosen since all biomarkers were measured at this timepoint.

### Outcome Definition

In this study, the CV outcome was defined as a composite of non-fatal myocardial infarction, non-fatal stroke, or cardiovascular death. The kidney outcome was defined as the composite of a confirmed doubling of serum creatinine (DSCR) or end-stage kidney disease (ESKD). In exploratory analyses, we also predicted the effect of empagliflozin on the composite heart failure hospitalization or CV death outcome and the composite kidney outcome of a sustained declined in estimated glomerular filtration rate (eGFR) by 40%, or ESKD. For the ongoing EMPA-KIDNEY trial, the primary outcome is defined as a composite of a sustained 40% decline in eGFR from baseline, ESKD, or kidney or CV death (Herrington et al., 2018).

### Statistical Analysis

A Cox proportional hazards model was fitted to estimate the coefficients associated with CV or kidney outcomes in the background dataset. These regression coefficients were then applied to the baseline and 6-months risk marker measurements for patients in the EMPA-REG OUTCOME trial, to estimate the risk for cardiorenal outcomes at both time points in both the placebo and empagliflozin arms, h(t) = h_o_(t) $\;e\sum^{\beta 1*X1}$ where the event rate at time t is a product of baseline hazard [h_o_(t)] and the sum of the linear form of the estimated *β* coefficients and the respective risk maker measurement(s) (*X*). The mean difference in the predicted risk in the empagliflozin arm, adjusted for the mean difference in their predicted risk in the placebo arm, represented the PRE score and reflected an estimation of the expected CV or kidney relative risk reduction (RRR) induced by empagliflozin treatment. To generate 95% confidence intervals (CI) on the predicted RRR, 100 sets of coefficients were generated from independent normal distributions based on the estimated regression coefficients and their standard error from the Cox proportional hazards model.

Since the observed changes in the EMPA-REG OUTCOME trial may differ from the actual risk marker changes in the EMPA-KIDNEY trial, we conducted simulations using a simulated range of empagliflozin induced responses on albuminuria, HbA1c, SBP, and body weight. These risk markers were simulated because they are important risk markers for the kidney and CV outcomes and were reduced by empagliflozin treatment. Simulations were performed by shifting the distribution of responses in these risk markers. We selected different proportions of patients with changes in these risk markers during empagliflozin treatment where response was defined as a reduction more or equal than the median risk marker changes from baseline.

To account for missing data, Multiple Imputations by Chained-Equation (using the R package “MICE”, version 3.11.0) was performed on all variables that had missing values. Imputations were performed by a predictive mean matching, a semi-parametric approach which replaces missing values according to a multivariable regression. (White et al., 2011). Covariate distributions were checked visually to ensure reasonably imputed values. Means and standard deviations are provided for variables with a normal distribution. For UACR, we used medians and interquartile ranges due to the skewed distribution. A log-transformation of UACR was done for entry in the Cox proportional hazards model. Categorical variables are reported as frequencies and percentages. Two-sided *p*-values <0.05 were considered statistically significant. All statistical analyses were conducted with R version 4.1.1 (R Project for Statistical Computing, http://www.r-project.org).

## Results

In the EMPA-REG OUTCOME trial, a total of 7020 patients were randomly assigned to receive empagliflozin (N = 4687) or placebo (N = 2333) and were included in the intention-to-treat population. The EMPA-REG OUTCOME trial participants were characterized by a high CV risk and generally low risk of complications due to kidney disease. At baseline, the mean HbA1c was 8.1%, SBP was 135.4 mmHg, and all participants had a CV disease history. Mean eGFR was 74.0 ml/min/1.73m^2^ and 1819 (25.9%) participants had an eGFR <60 ml/min/1.73m^2^. Median UACR was 17.7 mg/g, and 2811 (40.0%) patients had a UACR ≥30 mg/g. Demographic and clinical characteristics of the patients were well balanced between the treatment and placebo group (Table 1).

**TABLE 1:** Baseline characteristics of patients included in the background population and the EMPA-REG OUTCOME trial.

Characteristic	Background population (N = 6355)	EMPA-REG OUTCOME trial total population (N = 7020)	
		Placebo (N = 2333)	Treatment (N = 4687)
Age (years)	61.0 (9.0)	63.2 (8.8)	63.1 (8.6)
Female, N (%)	2128 (33.5)	653 (28.0)	1351 (28.8)
Race, N (%)			
Caucasian	3529 (55.5)	1678 (71.9)	3403 (72.6)
Black	569 (9.0)	120 (5.1)	237 (5.1)
Asian	1527 (24.0)	511 (21.9)	1006 (21.4)
Others	730 (11.5)	24 (1.0)	41 (0.9)
eGFR (ml/min/1.73m^2^)	49.8 (23.3)	73.8 (21.1)	74.2 (21.6)
Glycated hemoglobin (%)	8.1 (1.7)	8.1 (0.8)	8.1 (0.9)
Systolic BP (mmHg)	144.8 (20.0)	135.8 (17.2)	135.3 (16.9)
UACR (mg/g)	276.9 (54.5, 1193.9)	17.7 (7.1, 74.3)	17.7 (7.1, 71.6)
Weight (kg)	83.4 (19.8)	86.6 (19.1)	86.2 (18.9)
Hemoglobin (g/L)	128 (18.7)	137.2 (14.8)	137.3 (14.7)
HDL-cholesterol (mmol/L)	1.2 (0.4)	1.1 (0.3)	1.2 (0.3)
LDL-cholesterol (mmol/L)	3.1 (1.3)	2.2 (0.9)	2.2 (0.9)
Uric acid (umol/L)	404.6 (105.7)	359.0 (99.5)	356.0 (98.0)
Potassium (mmol/L)	4.6 (0.5)	4.6 (0.5)	4.5 (0.5)

For numerical variables which are normally distributed, data is presented as mean (SD). For UACR, with a skewed distribution, median (IQR) is presented. Categorical variables are presented as frequency (%). BP, blood pressure; UACR, urinary-albumin-creatinine-ratio; HDL, high-density-lipoprotein; LDL, low-density-lipoprotein. Estimated glomerular filtration rate (eGFR) was estimated according to the Modification of Diet in Renal Disease (MDRD) formula as per the EMPA-REG OUTCOME trial protocol.

### Short-Term Changes in Risk Markers

**FIGURE 1:**
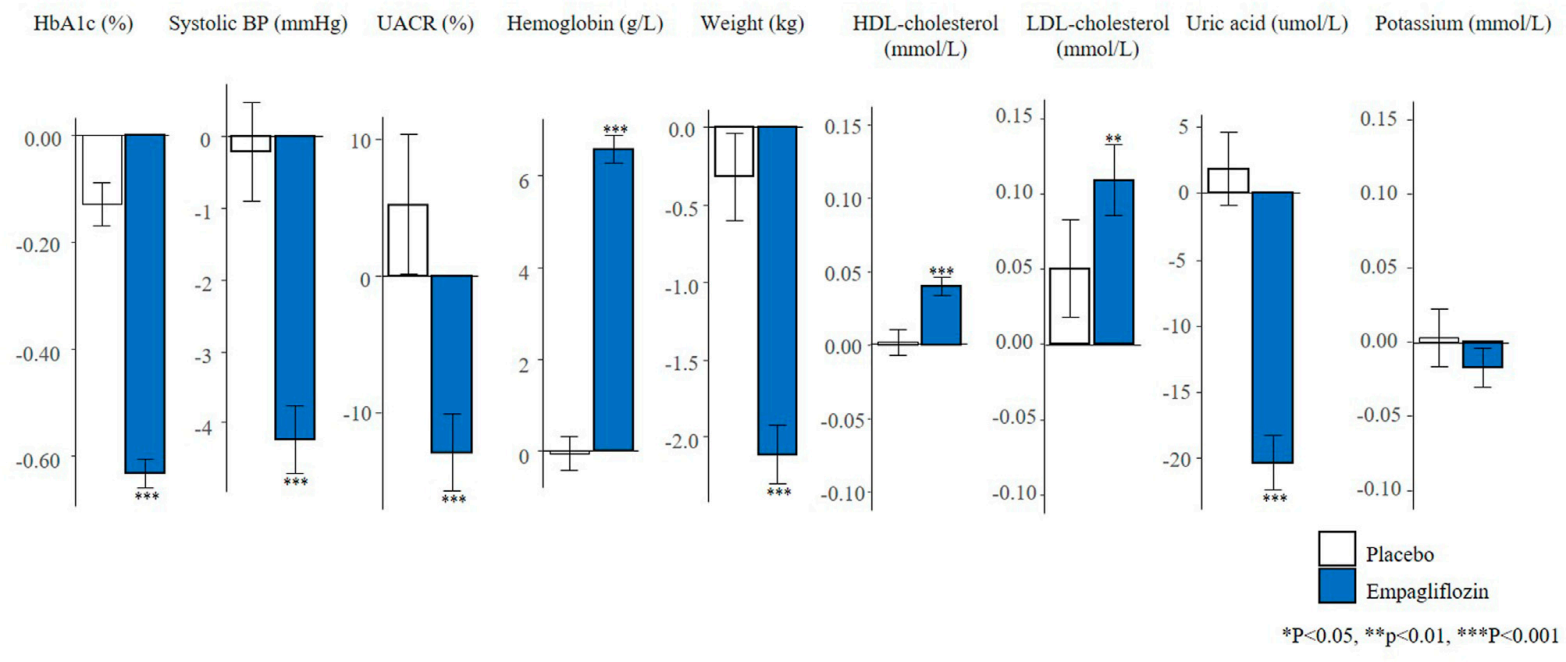
Mean changes in risk markers from baseline to 6-months in the total population of the EMPA-REG OUTCOME trial (N = 7020). Changes are presented as mean with 95% confidence interval, for the placebo and empagliflozin group. HbA1c, glycated hemoglobin; BP, blood pressure; UACR, urinary-albumin-creatinine-ratio; HDL, high-density-lipoprotein; LDL, low-density-lipoprotein.

**FIGURE 2:**
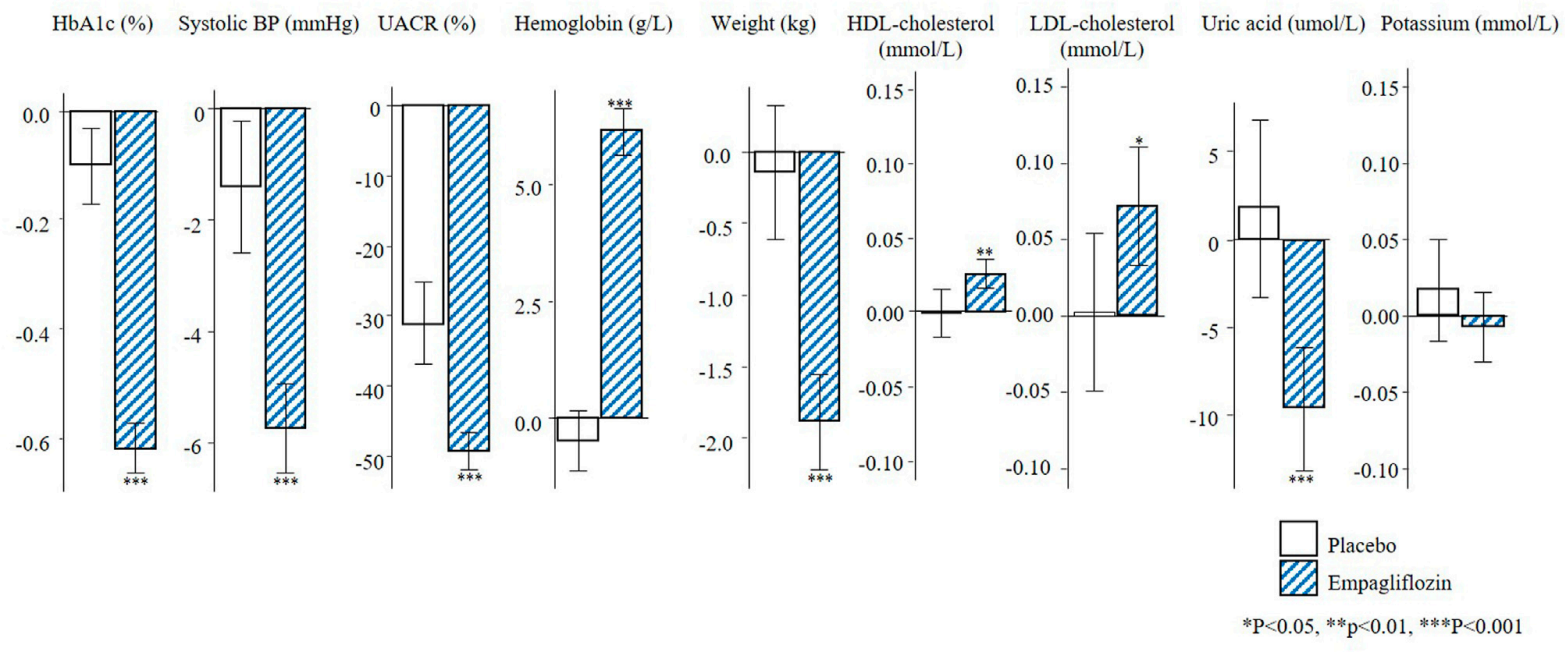
Mean changes in risk markers from baseline to 6-months in the population with UACR ≥30 mg/g of the EMPA-REG OUTCOME trial (N = 2811). Changes are presented as mean with 95% confidence intervals, for the placebo and empagliflozin group. HbA1c, glycated hemoglobin; BP, blood pressure; UACR, urinary-albumin-creatinine-ratio; HDL, high-density-lipoprotein; LDL, low-density-lipoprotein.

The changes in short-term risk markers for the total population and individuals with UACR >30 mg/g in the EMPA-REG OUTCOME trial are presented. Empagliflozin compared to placebo significantly reduced HbA1c (0.6%), SBP (4.2 mmHg), UACR (13.0%), body weight (2.1 kg), uric acid (20.4 umol/L) after 6 months of treatment, and increased hemoglobin (6.6 g/L), HDL-cholesterol (0.04 mmol/L), and LDL-cholesterol (0.1 mmol/L) (Figure 1). The direction and magnitude of the effect of empagliflozin in patients with UACR >30 mg/g (Figure 2) was generally similar as compared to the overall population. However, there was one exception. The reduction in UACR was more pronounced particularly in the empagliflozin group although in the placebo group UACR was also decreased possibly reflecting a regression to the mean.

### Observed and PRE Score Predicted Treatment Effect

**FIGURE 3:**
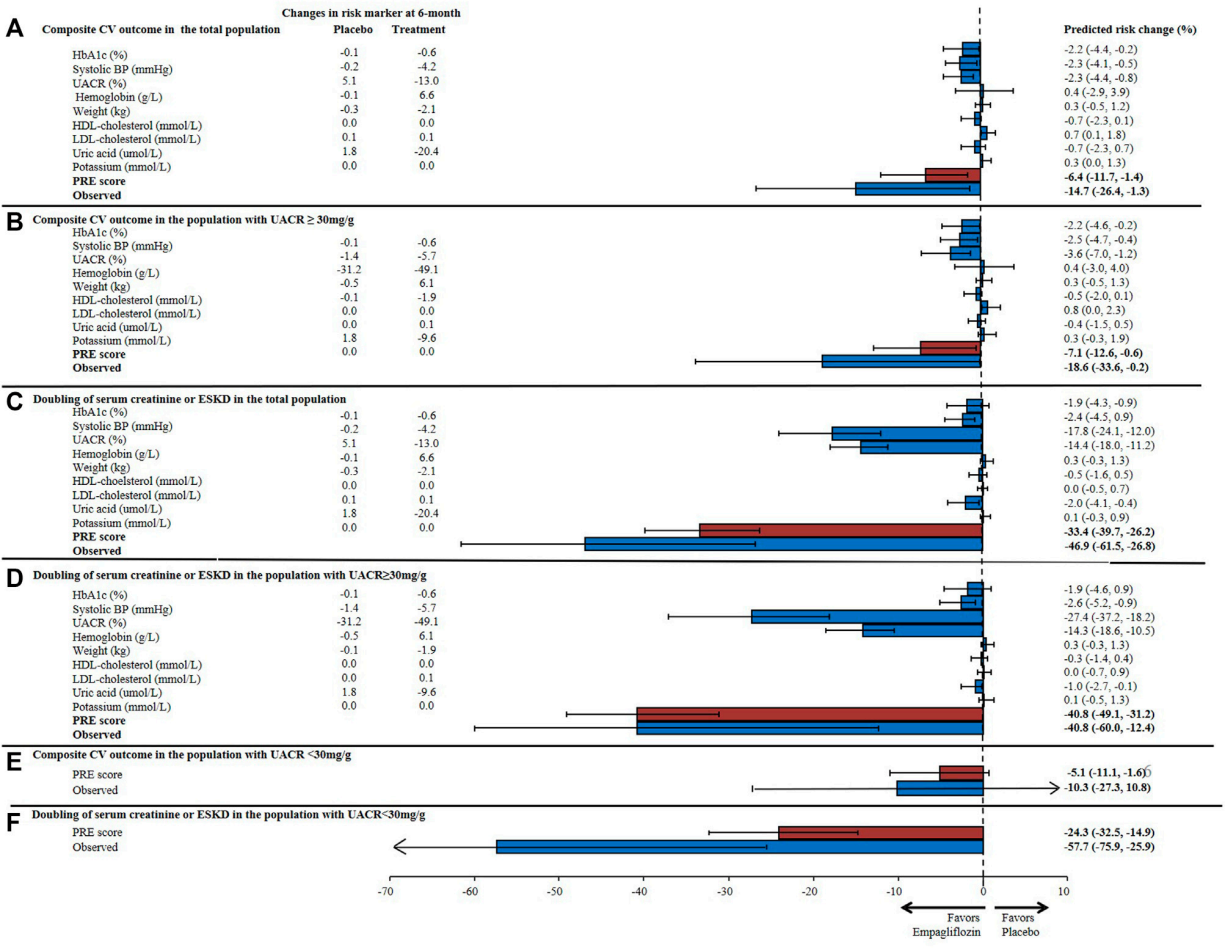
Predicted risk change for **(A)** the composite cardiovascular (CV) outcome consisting of non-fatal myocardial infarction, non-fatal stroke, or cardiovascular death in the total population; **(B)** the composite CV outcome in patients with UACR ≥30 mg/g; **(C)** the composite kidney outcome (doubling of serum creatinine or end-stage kidney disease) in the total population; and **(D)** the composite kidney outcome in patients with UACR ≥30 mg/g, **(E)** the composite CV outcome in patients with UACR <30 mg/g; **(F)** the composite kidney outcome in patients with UACR <30 mg/g based upon single and the multiple risk marker changes using the PRE score. Bars indicate the mean percentage reduction in the relative risk with 95% confidence intervals, following empagliflozin treatment. HbA1c, glycated hemoglobin; BP, blood pressure; UACR, urinary-albumin-creatinine-ratio; HDL, high-density-lipoprotein; LDL, low-density-lipoprotein; PRE score, Parameter Response Efficacy score.

During 3.1 years of follow-up, 772 (11.0%) composite CV outcomes, 150 (2.1%) composite kidney outcomes and 489 (7.0%) heart failure hospitalizations or CV deaths were recorded in the EMPA-REG OUTCOME trial. The observed RRR of empagliflozin on the composite CV outcome was 14.7% (95% CI 1.3–26.4) and the RRR for the composite kidney outcome was 46.9% (95% CI 26.8–61.5). The prediction of the treatment effect of empagliflozin based on single risk markers underestimated the overall treatment effect for the CV and kidney outcomes. For example, based on the observed placebo-corrected change in HbA1c alone, it was estimated that empagliflozin would reduce the risk of the composite CV outcome by 2.2% (95% CI 0.2–4.4) and the kidney outcome by 1.9% (95% CI 0.9–4.3). Based on the change in albuminuria alone, the predicted effect of empagliflozin on the CV outcome was 2.3% (95% CI 0.8–4.4) and 17.8% (95% CI 12.0–24.1) for the kidney outcome. Integrating changes in multiple short-term risk markers using the PRE score resulted in a predicted RRR for the CV outcome of 6.4% (95% CI 1.4–11.7) and for the kidney outcome of 33.4% (95% CI 26.2–39.7) (Figure 3). The predicted and observed RRR for the heart failure or CV death outcome and for the composite kidney outcome defined as 40% eGFR decline or end-stage kidney disease are reported in Supplementary Figure S1. The PRE score underestimated the effects of empagliflozin on both of the outcomes in the exploratory analyses.

In the subgroup of patients with UACR >30 mg/g, the mean HbA1c was 8.2% and SBP 140.0 mmHg at baseline. The mean eGFR was 70.2 ml/min/1.73m^2^ and there were 964 (34.3%) patients who had an eGFR <60 ml/min/1.73m^2^. The median UACR at baseline was 106.1 mg/g (Supplementary Table S2). The observed RRR for the composite kidney outcome in patients with UACR >30 mg/g was 40.8% (95% CI 12.4–60.0) and the PRE score predicted the same RRR of 40.8% (95% CI 31.2–49.1). The PRE score underestimated the observed treatment effect of empagliflozin in patients with baseline UACR <30 mg/g (Figure 2).

### Prediction of the EMPA-KIDNEY Outcomes

**FIGURE 4:**
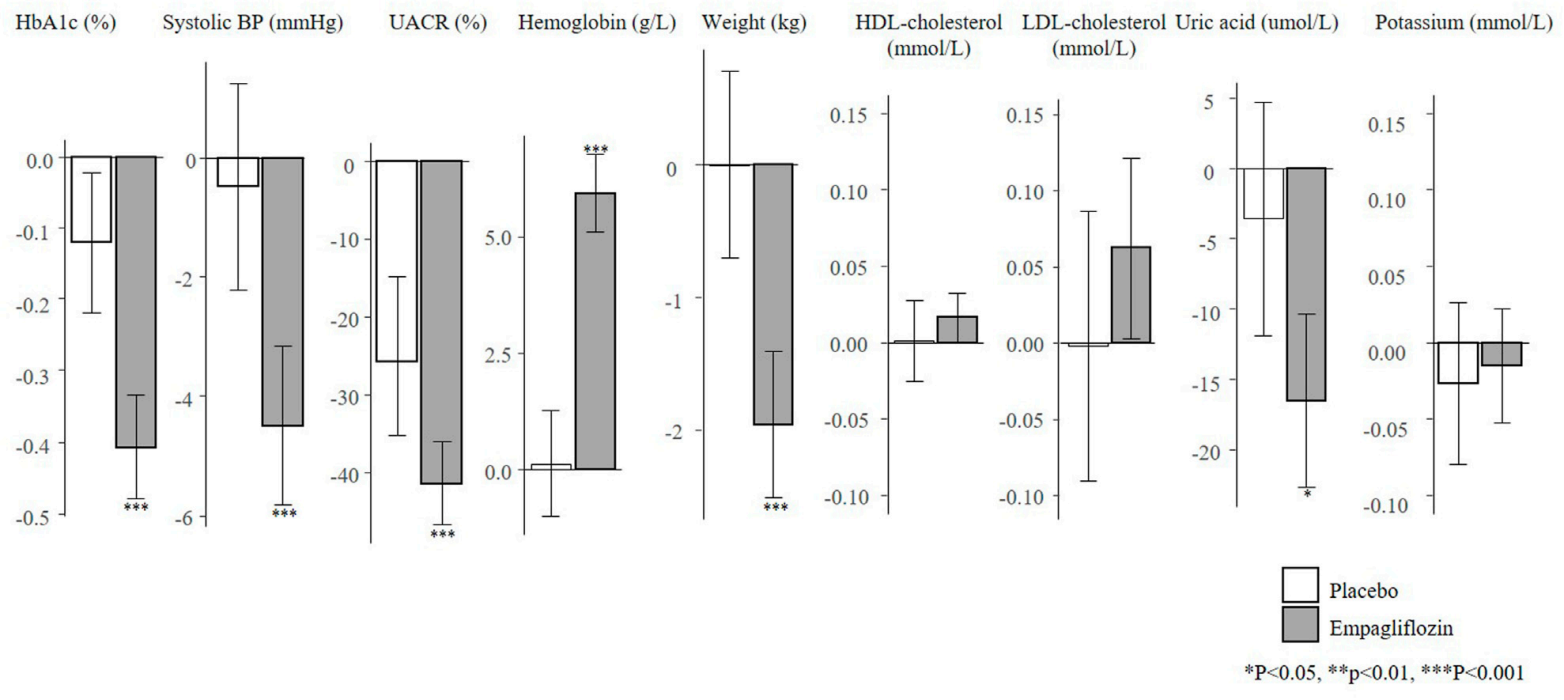
Mean changes in risk markers from baseline to 6-months in the subset of the EMPA-REG OUTCOME trial according to the EMPA-KIDNEY trial inclusion criteria (N = 1222). Changes are presented as mean with 95% confidence intervals, for the placebo and empagliflozin group. HbA1c, glycated hemoglobin; UACR, urinary-albumin-creatinine-ratio; BP, blood pressure; HDL, high-density-lipoprotein; LDL, low-density-lipoprotein.

There were 1222 (17.4%) patients from the EMPA-REG OUTCOME trial who met the inclusion criteria of the EMPA-KIDNEY trial. In this subgroup, the mean age was 65.8 years, mean eGFR was 52.9 ml/min/1.73m^2^ and median UACR was 360 mg/g (Supplementary Table S3). The baseline characteristics of these individuals were well balanced between the placebo and empagliflozin group. Empagliflozin reduced HbA1c (0.4%), SBP (4.5 mmHg), UACR (41.5%), body weight (2.0 kg), uric acid (16.5 umol/L), and increased hemoglobin (6.0 g/L) (Figure 4).

**FIGURE 5:**
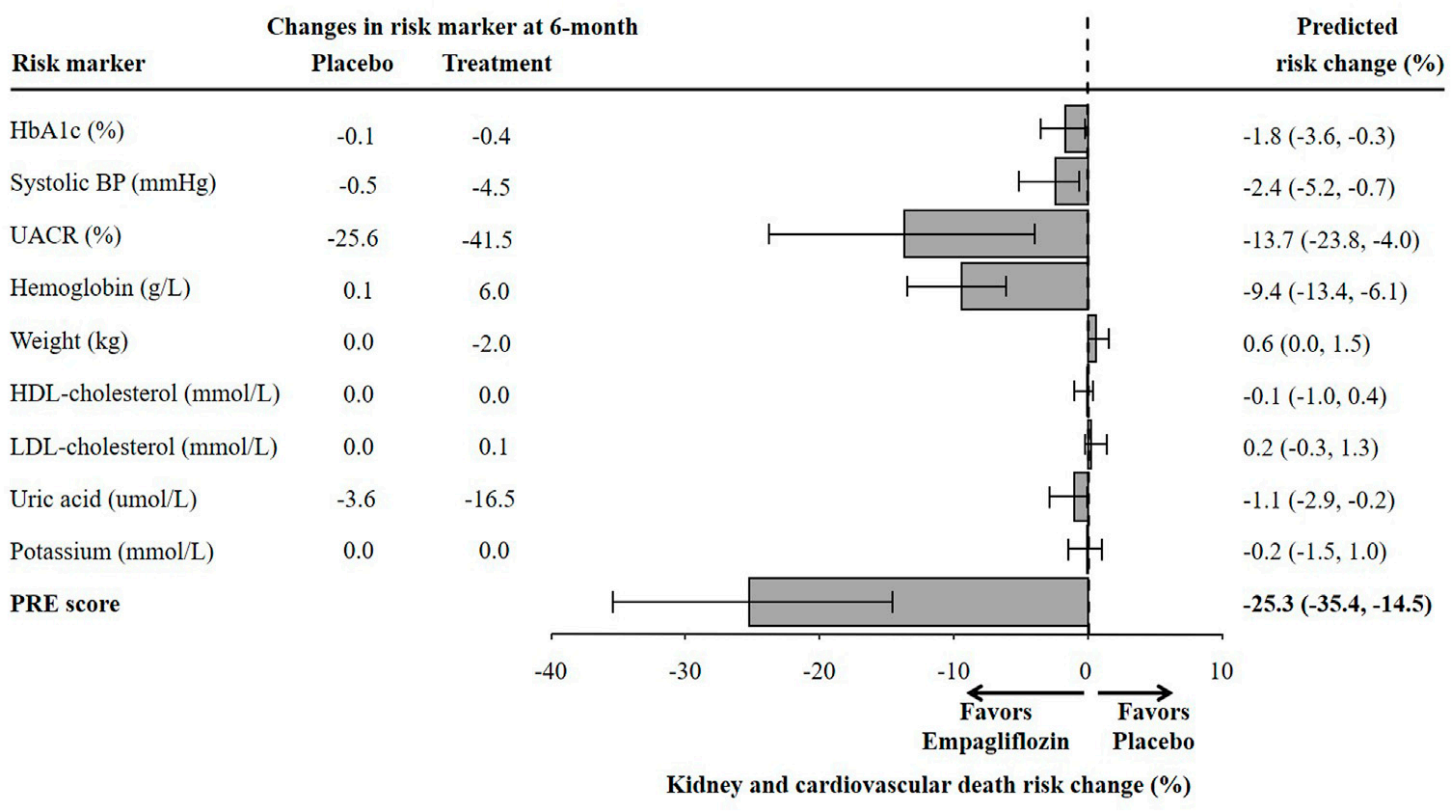
Predicted risk change for the composite kidney or cardiovascular death outcomes in the ongoing EMPA-KIDNEY trial based on single risk marker changes as well as the PRE score. The estimation of risk change was performed in a subset of the EMPA-REG OUTCOME trial population fulfilling the EMPA-KIDNEY trial inclusion criteria (N = 1222). HbA1c, glycated hemoglobin; BP, blood pressure; UACR, urinary-albumin-creatinine-ratio; HDL, high-density-lipoprotein; LDL, low-density-lipoprotein; PRE score, Parameter Response Efficacy score.

**FIGURE 6:**
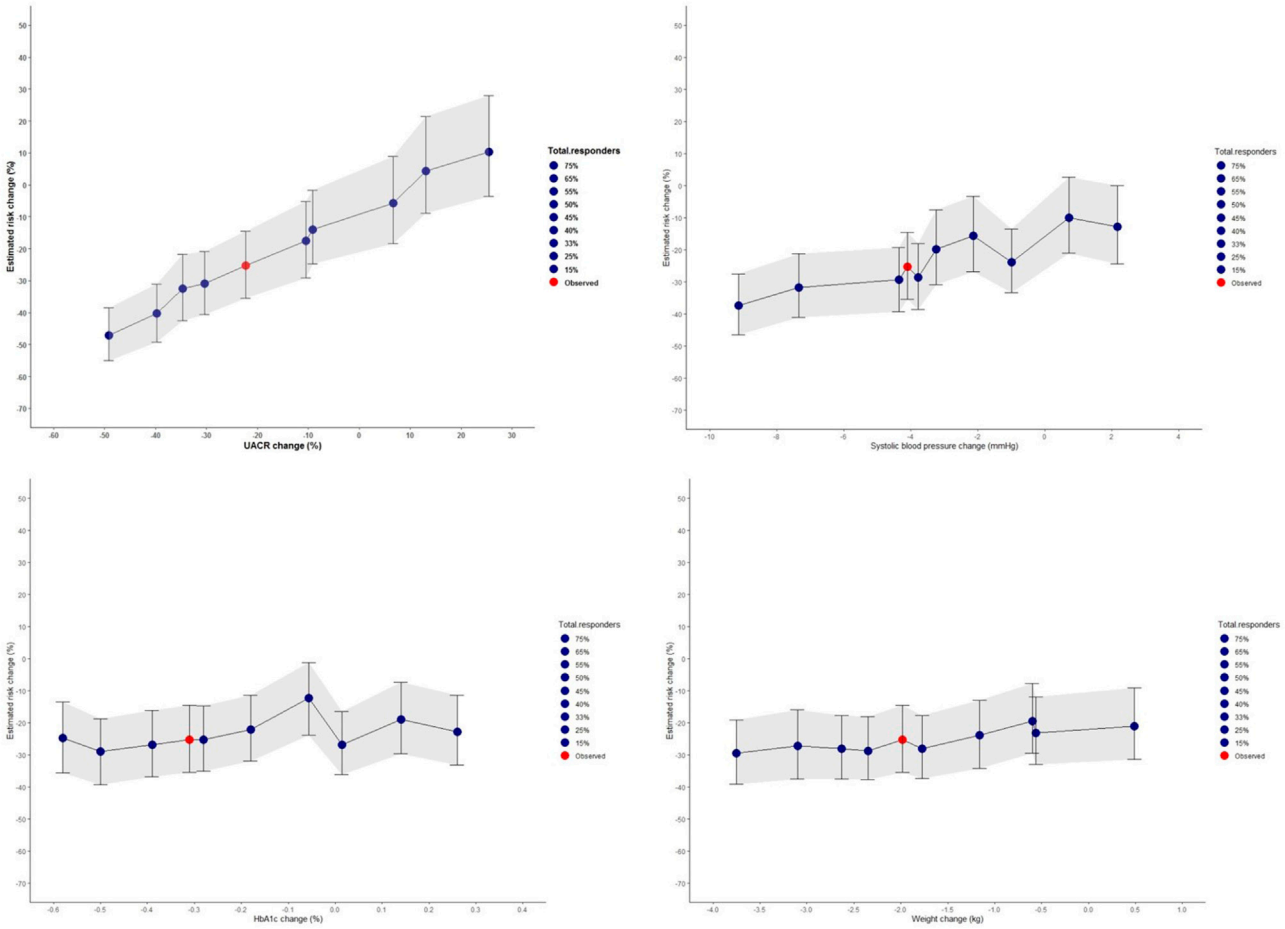
Simulated risk marker changes and the effect on the composite kidney and cardiovascular death outcomes for the prospective EMPA-KIDNEY trial based on the selected EMPA-REG OUTCOME trial population. The shaded area indicates the 95% confidence intervals for the simulated risk marker changes. Risk prediction was estimated from short-term risk marker changes in the EMPA-KIDNEY population (N = 1222) chosen from the EMPA-REG OUTCOME trial, and simulated values for UACR, systolic blood pressure, HbA1c, and body weight. The red dot indicates the PRE score predicted risk change in the EMPA-KIDNEY trial without enrichment with responders. The blue dots represent changes observed in the different proportion of responders and non-responders for each risk marker. HbA1c, glycated hemoglobin; UACR, urinary-albumin-to-creatinine ratio.

Using the PRE score, we estimated that empagliflozin, as compared to placebo, will lead to a RRR of 25.3% (95% CI 14.5–35.4) for the composite kidney or CV death outcome in the EMPA-KIDNEY trial (Figure 5). The simulation analyses revealed that the effect of empagliflozin was predominantly dependent on changes in UACR such that with larger effects of empagliflozin on UACR, the effect on kidney outcomes were markedly more pronounced. To achieve a RRR in the EMPA-KIDNEY trial outcomes by 25.3%, at least a 22.0% reduction in UACR will be needed (Figure 6).

## Discussion

In this study, we integrated multiple effects of the SGLT2 inhibitor empagliflozin on cardiorenal risk markers in a response efficacy score to predict the effect of empagliflozin on CV and kidney outcomes in the EMPA-REG OUTCOME trial. In the overall population, the PRE score underestimated the effect of empagliflozin on CV and kidney outcomes. However, in the subgroup analyses among individuals with UACR ≥30 mg/g, the PRE score accurately predicted the kidney outcome which was similar to the observed RRR achieved with empagliflozin.

The underestimation of the PRE score prediction suggests that the underlying mechanisms for CV and kidney protection are not fully captured by the clinical markers included in the current PRE score. The mechanism of action of SGLT2 inhibitors is complex and multifactorial and it is plausible that the set of readily available clinical markers cannot capture the multiple mechanistic pathways targeted by SGLT2 inhibitors (Heerspink et al., 2019; Sen and Heerspink, 2021). Indeed, studies have shown that SGLT2 inhibitors improve tubular oxygen tension, promote erythropoiesis through hypoxia-inducible factors, and improve cardiac metabolism and bioenergetics amongst others (Heerspink et al., 2016; Cowie and Fisher, 2020; Packer, 2021). Addition of biomarkers that specifically reflect these mechanistic pathways could improve efficacy prediction and could possibly be used as pharmacodynamic response biomarkers for SGLT2 inhibitors (Heerspink et al., 2019; Sen et al., 2021).

SGLT2 inhibitors were initially developed as oral glucose lowering drugs and approved by the regulatory agencies based on their HbA1c lowering effects (U.S. Food and Drug Administration, 2016). However, our analyses demonstrated that the reduction in HbA1c induced by empagliflozin largely underestimated the protective effect on CV and kidney outcomes. These results are in line with the results from other trials with SGLT2 inhibitors. In the EMPEROR-Reduced and EMPEROR-Preserved trials, empagliflozin reduced the risk of heart failure hospitalizations or CV death in heart failure patients with reduced or preserved ejection fraction regardless of diabetes status (Anker et al., 2021a; Anker et al., 2021b). Similarly, the benefits of dapagliflozin on clinical outcomes in patients with heart failure or chronic kidney disease were present independent of diabetes status (McMurray et al., 2019; Heerspink et al., 2020). Finally, in a head-to-head trial with glimepiride, the SGLT2 inhibitor canagliflozin slowed the decline in eGFR at equal glycemic control (Heerspink et al., 2017). Taken together, these findings suggest that the cardiorenal effect of SGLT2 inhibitors are not related to HbA1c changes alone and indicate the involvement of other non-glycemic pathways (Tuttle et al., 2021).

Albuminuria was a strong predictor of the effect of empagliflozin on kidney protective effects. Previous studies with the PRE score have also shown that the reduction in albuminuria was a strong contributor to the kidney protective effect of angiotensin receptor blockers, endothelin receptor antagonists and the SGLT2 inhibitor dapagliflozin (Smink et al., 2014b; Schievink et al., 2016; Idzerda et al., 2019). Interestingly, we showed that the effect of empagliflozin on kidney outcomes appeared to vary by baseline albuminuria. Among individuals with higher degree of albuminuria, the effect of empagliflozin was more pronounced and this effect was adequately predicted by the PRE score. Similar results have been observed in the CANVAS trial where the mediating effects of UACR were found to be significantly higher among patients with baseline UACR ≥30 mg/g, as compared to those with baseline UACR <30 mg/g (42 vs. 7%), and this suggests that the mechanism of SGLT2 inhibitors to protect the kidney may vary across patient subgroups (Li et al., 2020a). The early increase in hemoglobin was another important predictor of the benefit of empagliflozin. This finding is consistent with previous mediation analyses demonstrating that the change in hemoglobin explains a substantial proportion of the benefit of SGLT2 inhibitors on kidney and heart failure outcomes (Inzucchi et al., 2018; Li et al., 2020a; Li et al., 2020b). An increase in hemoglobin can be explained by the contraction in plasma volume as a result of SGLT2 inhibitor induced natriuresis and diuresis, but it can also reflect effects on hematopoiesis since SGLT2 inhibitors enhance erythropoietin production (Lambers Heerspink et al., 2013).

The EMPA-KIDNEY trial is the first dedicated kidney outcome trial with empagliflozin. The trial enrolled patients with chronic kidney disease with and without diabetes across a wide spectrum of eGFR and albuminuria values (Herrington et al., 2018). We estimated using the observed biomarker changes that empagliflozin will reduce the primary kidney outcome in EMPA-KIDNEY by 25%. We note, however, that these predictions are conditional on the actual population enrolled in the EMPA-KIDNEY trial and the actual observed risk marker changes. Our simulation analyses showed that if the effects of empagliflozin on UACR in the EMPA-KIDNEY population are larger than those observed in the EMPA-REG OUTCOME trial it may possibly result in a larger RRR of the primary kidney outcome. The ongoing EMPA-KIDNEY trial will soon deliver the answer whether our prediction of the treatment effect of empagliflozin was accurate.

There are several limitations in this study. First, the selection of parameters in the PRE score depends on the availability of the risk markers in the trial dataset. Second, the majority of the patients included in the EMPA-REG OUTCOME trial had mild to moderate chronic kidney disease and only few patients met the entry criteria for the EMPA-KIDNEY trial. As a result, the prediction of the EMPA-KIDNEY endpoint is based on a limited sample size and low precision. Furthermore, we note that the EMPA-KIDNEY trial enrolled patients with and without diabetes and our predictions only apply to diabetes patients since patients without diabetes were not included in the background population nor the EMPA-REG OUTCOME trial. Nevertheless, given the consistent effects of SGLT2 inhibitors in patients with and without diabetes this should not affect our findings (Wheeler et al., 2021).

To conclude, the PRE score underestimated the treatment effect of empagliflozin on CV and kidney outcomes in patients with type 2 diabetes and established CV disease. Whether the addition of novel biomarkers, which capture mechanistic pathways targeted by empagliflozin, to the PRE score improves treatment effect predictions requires further study.

## Data Availability

The original contributions presented in the study are included in the article/Supplementary Material, further inquiries can be directed to the corresponding author.
