# Supplementary material for: Prediction of the Effects of Empagliflozin on Cardiovascular and Kidney Outcomes Based on Short-Term Changes in Multiple Risk Markers
Source: Front Pharmacol. 2022 Jan 25;12:786706. doi: 10.3389/fphar.2021.786706 (PMC8821652; doi:10.3389/fphar.2021.786706)

Supplementary Material

**Supplemental** **Table 1.** Description of studies included as background dataset and the EMPA-REG OUTCOME trial dataset

| Study name and ClinicalTrials.gov identifier | Study description | Median follow-up time (years) | Intervention | No. of patients included in analysis/ Total patients enrolled in the trial |
| --- | --- | --- | --- | --- |
| *Studies included in the background dataset (N=6355)* | | | | |
| RENAAL [NCT00308347] | RCT among people with non-insulin dependent diabetes mellitus, hypertension, and urinary protein>1+ on dipstick | 3.4 | Losartan (50 to 100 mg once daily) or placebo | 1471/1513 |
| IDNT [NCT00317915] | RCT among people with diabetes mellitus, hypertension, and nephropathy | 2.6 | Irbesartan (300 mg daily), amlodipine (10 mg daily), or placebo | 1703/1715 |
| ALTITUDE  [NCT00549757] | RCT among people with diabetes mellitus, with evidence of albuminuria and history of cardiovascular disease | 2.7 | Aliskiren 300 mg/day, or placebo as adjunct to ACEi/ARB | 3181/8561 |
| *The EMPA-REG OUTCOME trial dataset (N=7020)* | | | | |
| EMPA-REG Outcome [NCT01131676] | RCT in patients with diabetes mellitus, at high risk for cardiovascular events | 3.1 | Empagliflozin 10mg or 25mg  versus placebo | 7020/7020 |

# RENAAL, Reduction of Endpoints in NIDDM with the Angiotensin II Antagonist Losartan; IDNT, Irbesartan Diabetic Nephropathy Trial; ALTITUDE, Aliskiren Trial in Type 2 Diabetes Using Cardiorenal Endpoints; EMPA-REG OUTCOME, Empagliflozin Cardiovascular Outcome Event Trial in Type 2 Diabetes Mellitus Patients. RCT, randomized controlled trial; ACEi, angiotensin converting enzyme inhibitors; ARB, angiotensin II receptor blocker.

**Supplemental** **Table 2.** Baseline characteristics of the background population and the subgroup of patients from the EMPA-REG OUTCOME trial with UACR ≥30 mg/g

| **Characteristic** | **Background population**  **(N=6355)** | **EMPA-REG OUTCOME Trial population with UACR** ≥**30 mg/g** | | |
| --- | --- | --- | --- | --- |
|  |  | **Total**  **(N=2811)** | **Placebo**  **(N=944)** | **Treatment**  **(N=1867)** |
| Age (years) | 61.0 (9.0) | 63.9 (8.6) | 63.8 (8.6) | 64.0 (8.6) |
| Female, n(%) | 2128 (33.5) | 714 (25.4) | 233 (24.7) | 481 (25.8) |
| Race, n(%) |  |  |  |  |
| Caucasian | 3529 (55.5) | 1933 (68.8) | 652 (69.1) | 1281 (68.6) |
| Black | 569 (9.0) | 143 (5.1) | 55 (5.8) | 88 (4.7) |
| Asian | 1527 (24.0) | 703 (25.0) | 226 (23.9) | 477 (25.5) |
| Others | 730 (11.5) | 32 (1.1) | 11 (1.2) | 21 (1.1) |
| eGFR (ml/min/1.73m^2^) * | 49.8 (23.3) | 70.2 (22.0) | 70.0 (21.5) | 70.3 (22.3) |
| Glycated hemoglobin (%) | 8.1 (1.7) | 8.2 (0.9) | 8.2 (0.9) | 8.2 (0.9) |
| Systolic BP (mmHg) | 144.8 (20.0) | 140.0 (17.9) | 140.1 (18.2) | 140 (17.7) |
| UACR (mg/g) | 276.9 [54.5, 1193.9] | 106.1 [52.2, 353.6] | 108.7 [51.3, 363.5] | 105.2 [52.2, 351.8]] |
| Weight (kg) | 83.4 (19.8) | 85.7 (19.2) | 86.4 (19.1) | 85.3 (19.2) |
| Hemoglobin (g/L) | 128 (18.7) | 136.8 (15.8) | 136.5 (16.1) | 137.0 (15.6) |
| HDL-cholesterol (mmol/L) | 1.2 (0.4) | 1.1 (0.3) | 1.1 (0.3) | 1.2 (0.3) |
| LDL-cholesterol (mmol/L) | 3.1 (1.3) | 2.3 (1.0) | 2.3 (1.0) | 2.3 (1.0) |
| Uric acid (umol/L) | 404.6 (105.7) | 371.7 (103.3) | 377.0 (103.9) | 369.1 (103.0) |
| Potassium (mmol/L) | 4.6 (0.5) | 4.6 (0.5) | 4.6 (0.5) | 4.6 (0.5) |

For numerical variables which are normally distributed, data is presented as mean (SD). For urinary-albumin-creatinine-ratio (UACR) with a skewed distribution, median [IQR] is presented. Categorical variables are presented as frequency (%). BP, blood pressure; HDL, high-density-lipoprotein; LDL, low-density-lipoprotein. *The estimated glomerular filtration rate (eGFR) was estimated according to the Modification of Diet in Renal Disease formula (MDRD) in the EMPA-REG OUTCOME trial protocol.

**Supplemental** **Table 3.** Baseline characteristics of patients in the EMPA-REG OUTCOME trial dataset whom fulfilled the inclusion criteria of the EMPA-KIDNEY trial (N=1222)

| **Characteristic** | **Subset of the EMPA-REG OUTCOME trial population with eGFR >20 to <45 mL/min/1.73m2, or eGFR ≥45 to <90 mL/min/1.73m² with UACR >200 mg/g** | | |
| --- | --- | --- | --- |
|  | **Total**  **(N=1222)** | **Placebo**  **(N=421)** | **Treatment**  **(N=801)** |
| Age (years) | 65.8 (8.2) | 65.5 (8.3) | 66.0 (8.2) |
| Female, n(%) | 364 (29.8) | 124 (29.5) | 240 (30.0) |
| Race, n(%) |  |  |  |
| Caucasian | 845 (69.1) | 289 (68.6) | 556 (69.4) |
| Black | 56 (4.6) | 21 (5.0) | 35 (4.4) |
| Asian | 304 (24.9) | 106 (25.2) | 198 (24.7) |
| Others | 17 (1.4) | 5 (1.2) | 12 (1.5) |
| eGFR (ml/min/1.73m^2^)* | 52.9 (16.3) | 53.1 (15.7) | 52.7 (16.5) |
| Glycated hemoglobin (%) | 8.2 (0.9) | 8.2 (0.9) | 8.1 (0.9) |
| Systolic BP (mmHg) | 139.8 (18.3) | 140.0 (18.7) | 139.7 (18.0) |
| UACR (mg/g) | 360.2 [61.4, 870.3] | 358.0 [72.5, 835.4] | 360.7 [61.4, 870.3] |
| Weight (kg) | 85.3 (19.3) | 85.3 (19.1) | 85.2 (19.4) |
| Hemoglobin (g/L) | 132.0 (16.7) | 132.0 (17.3) | 132.0 (16.5) |
| HDL-cholesterol (mmol/L) | 1.1 (0.3) | 1.1 (0.3) | 1.1 (0.3) |
| LDL-cholesterol (mmol/L) | 2.3 (1.0) | 2.3 (1.0) | 2.3 (1.0) |
| Uric acid (umol/L) | 413.3 (109.0) | 417.5 (103.2) | 411.0 (112.0) |
| Potassium (mmol/L) | 4.6 (0.5) | 4.6 (0.6) | 4.6 (0.5) |

For numerical variables which are normally distributed, data is presented as mean (SD). For urinary-albumin-creatinine-ratio (UACR) with a skewed distribution, median [IQR] is presented. Categorical variables are presented as frequency (%). BP, blood pressure; HDL, high-density-lipoprotein; LDL, low-density-lipoprotein. *The estimated glomerular filtration rate (eGFR) was estimated according to the Modification of Diet in Renal Disease formula (MDRD) in the EMPA-REG OUTCOME trial protocol.

**Supplemental** **Figure 1.** The observed and predicted risk change for hospitalization for the heart failure or cardiovascular death endpoint in (A) the total population and (B) patients with UACR ≥30 mg/g in the EMPA-REG OUTCOME trial are shown, based upon single and the multiple risk marker changes using the PRE score. In the same figure, kidney outcomes according to a sustained declined in eGFR by 40% or end stage kidney disease (ESKD) in (C) the total population and (D) patients with UACR ≥30 mg/g in the EMPA-REG OUTCOME are illustrated. Bars indicate mean relative risk reduction with 95% confidence intervals following empagliflozin treatment. HFCVD, heart failure or cardiovascular death; HbA1c, glycated hemoglobin; UACR, urinary-albumin-creatinine-ratio; BP, blood pressure; HDL, high-density-lipoprotein; LDL, low-density-lipoprotein; PRE score, Parameter Response Efficacy score.


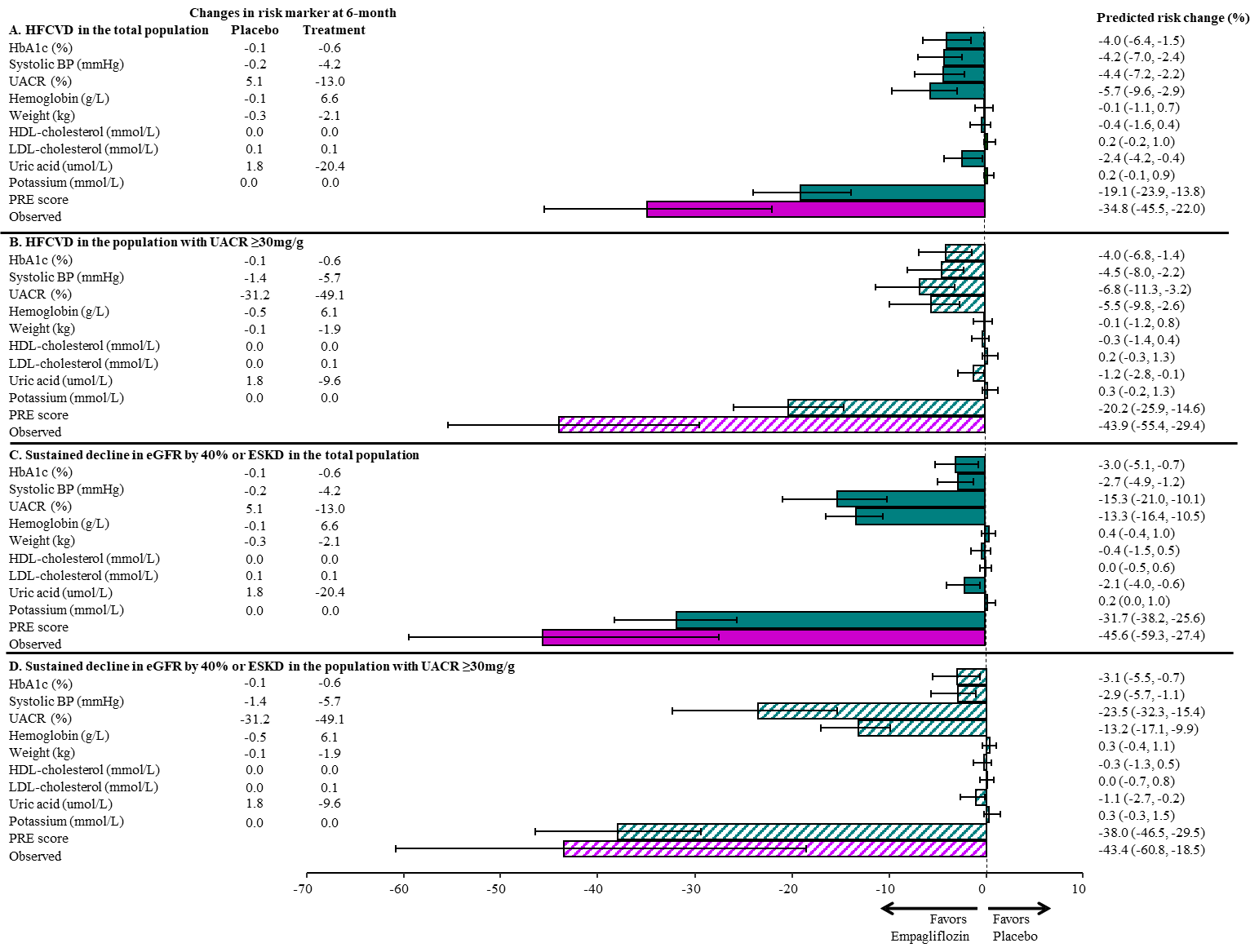

Supplement: Supplementary file 1 [file DataSheet1.docx]
